# Fear Conditioning With Film Clip and Electric Shock Unconditioned Stimuli: What Drives Conditioned Electrodermal Responses?

**DOI:** 10.1111/psyp.70089

**Published:** 2025-06-10

**Authors:** Lilyan Tyson, Ruqayya Dawoodjee, Joe Anderson, Ottmar V. Lipp, Gia Nhi Lam, Kalia White, Jack Cooper, Luke J. Ney

**Affiliations:** ^1^ School of Psychology and Counselling Faculty of Health, Queensland University of Technology Brisbane Queensland Australia

**Keywords:** fear conditioning, intrusive memory, skin conductance response, unconditioned stimulus

## Abstract

When compared to audio or video stimuli, previous studies have shown that electric shocks produce stronger electrodermal conditioned fear responses. This difference occurs even if the audio or video stimuli are perceived as more intense and aversive by participants. We re‐analyzed two datasets that used a combined trauma film/fear conditioning paradigm to test whether this effect was due to trace conditioning or other factors. We found evidence that weaker electrodermal conditioning when the unconditioned stimulus (US) is a film clip is unlikely to be due to trace conditioning, as no prominent delayed unconditioned responses occurred during the film clip after conditioned stimulus offset. We also found that some film clips elicit unique unconditioned responses, which may depend on the specific timing of the most aversive events within each clip. Finally, we found evidence that larger skin conductance responses during the presentation of one of the four trauma film clips used was associated with more frequently reported intrusive memories. This data provides important new information supporting the idea that skin conductance responses during conditioning using electric shock USs might not reflect purely anticipatory fear, at least in the way conceptualized in the literature. This account has potentially critical implications for the interpretation of fear conditioning research using electric shocks.

## Impact Statement

This article shows that weaker electrodermal fear conditioning when the unconditioned stimulus is an aversive film clip, compared to a shock, is not due to trace conditioning. This suggests that electrodermal responses during conditioning using electric shock USs might not reflect purely anticipatory fear, impacting the interpretation of fear conditioning research.

Fear conditioning is an experimental paradigm that is integral to our understanding of learning processes that are believed to be involved in traumatic and anxiety disorders (Bouton et al. 2020; Lonsdorf et al. 2017; Zuj and Norrholm 2019). Fear conditioning paradigms involve repeatedly pairing a neutral stimulus (a conditioned stimulus, or CS+) with an aversive outcome (an unconditioned stimulus, or US), such as a mild electric shock or an unpleasant noise. Repeated pairing of the two stimuli results in the previously neutral stimulus (the CS+) acquiring negative valence and becoming a predictive cue for something aversive. Fear conditioning paradigms have a long tradition of being used to explore fear learning mechanisms that might underlie anxiety disorders and traumatic disorders—such as posttraumatic stress disorder (PTSD), and have led to insights into how these disorders arise and are maintained over time (Craske et al. 2014; Lipp 2006).

Fear conditioning paradigms have often been critiqued for using USs that do not accurately represent the complexity of real‐world stressful events (Beckers et al. 2013). Therefore, the trauma film conditioning paradigm was created, which uses violent film clips as USs rather than conventional USs such as an electric shock (Wegerer et al. 2013). Trauma film conditioning paradigms involve repeated presentations of a CS+ followed by an aversive film clip (US) during acquisition, with both typical outcome measures of fear conditioning (e.g., skin conductance response) as well as intrusive memories of the trauma film clips assessed concurrently. Wegerer et al. (2013) reported that this paradigm allowed exploration of cues that elicit intrusive memories as re‐exposing participants to the CS cued intrusive memories relating to the film clips (Franke et al. 2021). The findings from these studies support the theory that neutral stimuli witnessed before a traumatic event develop into contextual cues that may trigger intrusive memories of the traumatic event (Ehlers and Clark 2000), opening the possibility to explore intrusive re‐experiencing in PTSD‐like paradigms with greater precision and validity (Franke et al. 2021; Ney, Schenker et al. 2022).

It is well established that differences in skin conductance responses (SCRs) during conditioning are observable when the nature of the CS+ differs in type and valence—for example, use of angry and fearful face CSs results in impaired fear extinction compared to both neutral face and shape CSs (Ney, Luck, et al. 2022; Ney, O'Donohue et al. 2022). Choice of US type also affects electrodermal fear conditioning. For instance, auditory USs elicit smaller SCRs compared to electro‐tactile USs presented alone or in combination with auditory USs during standard fear conditioning, in response to the CS as well as the US itself (Ney, Nichols et al. 2023). Evidence of similar differences has been observed in the trauma film conditioning paradigm. While participants in these paradigms tend to rate the film clips as more aversive than electric shocks and show equivalent conditioning in subjectively rated outcome measures (Ney, Cooper et al. 2023), meta‐analysis as well as empirical studies have found that SCRs are lower to CSs + paired with film clip USs alone compared to CSs + paired with electric shocks alone or CSs + paired with film clips and electric shocks presented simultaneously (Franke et al. 2022; Ney, Schenker et al. 2022).

Why this difference occurs has not been identified, though there are several possible explanations. Firstly, the duration of the clips may contribute to participants' differing physiological responses. The duration of the film clips is typically 16 s, whereas the shock lasts only 50–500 milliseconds. The physiological response to the clip may be elongated for the duration of the clip and thus less time locked to its onset compared to the response to the shorter shock. This explanation is supported by previous evidence showing that a longer CS+ duration results in weaker conditioning (Hinchy et al. 1995). If the most aversive events in a 16 s film clip occur in the later part of the clips, then the relatively innocuous start of the clip may become an extended part of the CS+, hence extending the CS+ duration and weakening conditioning.

Another explanation for the difference in conditioned SCRs observed across paradigms is whether delay or trace conditioning mediates fear acquisition. In delayed conditioning, the onset of the US occurs during or coinciding with the offset of the CS without a temporal gap, whereas in trace conditioning the US is presented after a temporal gap after the offset of the CS (Lonsdorf et al. 2017; Rescorla 1988). Temporal contiguity plays a vital part in fear conditioning as it has been found that the development of fear acquisition and extinction occurs more rapidly in delay than trace conditioning (Beylin et al. 2001; Ewald et al. 2014; Knight et al. 2004; Lipp et al. 2003; Rescorla 1988). By extension, this implies that if the most aversive part of a violent clip US has a temporal delay after the offset of the CS, conditioning will be weaker. Indeed, it is well established that physiological and behavioral responses directly to film clips depend on the content of the clip that is presented, including the timing of the most intense events in the films (Bos et al. 2013; Kreibig et al. 2007; Mauss et al. 2005).

Alternatively, the difference in physicality between the types of USs may contribute to the observed differences in SCR. To satisfy a diagnosis for PTSD, the DSM‐5 requires that the traumatic event was actually harmful or was perceived to be physically harmful and life‐threatening to oneself, a family member, or a close friend (American Psychiatric Association 2013). Under this definition of threat, the exposure to the aversive event must be extreme, repeated, or involving possible physical harm; however, this does not apply to exposure to electronic media such as film clips unless this media is work related (American Psychiatric Association 2013). A mild electric shock in an unfamiliar environment may be perceived as a potential real‐world physical threat by participants naïve to the actual safety of fear conditioning procedures. It is therefore possible that electric shocks produce a distinct physical, pain, or autonomic response that differs from nonphysical and purely psychological stimulation through an implicit and automatic reflexive response that is outside of a participant's awareness or control.

The current study presents a re‐analysis of two studies using the combined trauma film conditioning paradigm. Participants were exposed to conditioning with CS + s followed by either violent video clips alone or violent video clips as well as mild electric shocks. This data is either under review or published (Lam et al. 2024) and we found in these publications evidence of weak SCR conditioning to film clips compared to shock USs, though higher subjective aversiveness ratings for the film clip USs and equivalent conditioning between film clips and shock USs when measured by self‐reported CS pleasantness. In the current re‐analysis, we tested (1) whether different types of video clips resulted in different SCR patterns during video US presentation, (2) whether the addition of a shock at clip onset changed electrodermal response patterns during video US presentation, and (3) whether SCRs to specific video clips were correlated with the number of reported intrusive memories that had content matching those clips.

These hypotheses were based on the idea that trace conditioning could be the cause of lower conditioned responding when USs are used as film clips. If this were the case, then we would see prominent unconditioned responses later in the US interval during film clip only USs compared to USs including shocks. We hypothesized that (1) different video clips would elicit unique SCR patterns, (2) larger SCRs would be observed in the presence of a shock US, and (3) higher SCRs to specific video clips would be associated with higher intrusive memory frequency for that video clip content. If hypothesis (1) was correct, then we could correlate the delayed unconditioned responses during film clips with specific events in those clips. This would allow us to understand whether delayed unconditioned responses were occurring during the film clips. If delayed responses were occurring, then we could infer that trace conditioning may be the cause of reduced conditioned responding. If hypothesis (2) was correct (irrespective of hypothesis 1 outcome), then we could infer that shock administration creates larger USs, which could explain lower conditioned responses in film clip only conditions, irrespective of US timing. If hypothesis (3) was correct, we could infer that the magnitude of the US response was associated with the likelihood of intrusive memory development. Testing these hypotheses allowed us to assess a number of factors involved in the relationship between conditioned responding, US type and timing, and intrusive memories.

## 1 Experiment 1

### 1.1 Methods

Experiment 1 used a conditioned intrusion paradigm that featured a within‐subject manipulation, where a CS+ presented during acquisition was followed by a 16‐s film clip and a shock, while another CS+ was followed only by the film clip. This experiment is a re‐analysis of data reported in Lam et al. (2024).

### 1.2 Participants

Ethical approval was ascertained through the Queensland University of Technology (QUT) Human Research Ethics Committee. Experiment 1 involved the recruitment of participants through undergraduate research participation schemes, word‐of‐mouth, and community boards. Exclusion criteria, assessed through an online Qualtrics survey, included psychological diagnoses, cardiac, neurological, or epileptic conditions, current pregnancy, and/or a medical history of serious head injuries, that is, concussions. Those who reported medication usage were retained if they did not disclose physical or psychological disorders; however, as most participants were of good health, there were few using regular medications. Daily exposure to violent video games, films, or television series was an additional exclusion criterion. GPower 3.1.9.4 was used to ascertain a power calculation, with 2 groups (i.e., one between‐subjects factor with two levels and one within subjects' factor with three levels), estimated effect size of *f* = 0.15 (drawn from previous research in SCR and subjective fear ratings in fear conditioning (Landkroon et al. 2019; Wegerer et al. 2013)), alpha = 0.05, and power = 0.80. From this, an ideal sample size of 74 was produced; however, 99 participants were recruited to account for data loss, which included 21 males, 77 females, and 1 non‐binary participant (*M*_age_ = 21.16, SD = 5.50). The power calculation was performed to assess a renewal group manipulation described in Lam et al. (2024).

### 1.3 Materials and Measures

#### 1.3.1 Trauma Film

The trauma film comprised seven clips from commercial horror films, public safety campaigns, and news media, each lasting between one and three minutes and running for 10 min once combined. The two news segments were drawn from U.S. and Indian news channels to lower the chance of prior exposure to the content and covered a violent mugging and a leopard attack. The two safety campaign clips depicted accidental trauma, including Peter Watkins‐Hughes' “Cow” (2009), a texting and driving awareness advertisement, and the Workplace Safety and Insurance Board (2007) kitchen safety campaign, which has been used in previous research (Dibbets et al. 2018). The final three clips were drawn from horror films Nicolas Winding Refn's “Drive” (2011), Eli Roth's “Hostel” (2005), and “Irreversible” (2002) directed by Gaspar Noé, which depict gross interpersonal violence. The two latter movies have been utilized across prior research using trauma film material (Franke et al. 2021; Herzog et al. 2022; Wegerer et al. 2013).

#### 1.3.2 Neutral Film Clips

Neutral film clips were ascertained from the “Mixed emotions film library,” a resource curated for researchers to improve outcomes (Samson et al. 2016). The four 16‐s scenes were paired with the conditioned safety stimulus (CS−) during the experiment. The clips included a man shoveling snow (mean rating of neutrality of the film clip on scale 1–6 where 6 was “very strongly neutral” [*M*_neutrality_ = 4.46]), people in a subway train (*M*_neutrality_ = 4.51), a man riding a horse (*M*_neutrality_ = 4.23), and people sitting at a café (*M*_neutrality_ = 4.96).

#### 1.3.3 Fear Conditioning

##### 1.3.3.1 Unconditioned and Conditioned Stimuli

The fear paradigm utilized in Experiment 1 included habituation, acquisition, extinction, context renewal, and reacquisition, though only responses during acquisition are examined in this study, as this is when the different USs were presented. CSs consisted of geometric shapes, namely triangles, circles, and squares, with two CS+ and one CS− used in the experiment. Counterbalancing was used to minimize order and carryover effects. A Digitimer DS7A, an electric shock device, and a concentric shock electrode were used to generate the electric shock, which was paired with only one CS+ (the CS_1_+). Only four 16 s clips from the most aversive parts of the longer trauma film, specifically the “Hostel” and “Irreversible” scenes, along with two public safety campaigns (“Cow” and kitchen safety), were utilized as USs to be paired with both the CS_1_+ and CS_2_+. CS+ and CS− presentation was counterbalanced, with CS+ occurring on the first trial of acquisition for 50% of participants.

##### 1.3.3.2 Contextual Renewal

The fear conditioning task involved a context shift, where the habituation, acquisition, renewal, and reacquisition phases were presented in context A, whereas extinction was presented in context B (Wang et al. 2024). The contexts were counterbalanced and included either pink (RGB 255128192) or blue (RGB 0128255) colored backgrounds (simple background condition), or a leafy street or office background (complex background condition). We previously found no difference between these conditions during conditioning (Lam et al. 2024), so these effects are examined no further in this article.

##### 1.3.3.3 Equipment and Skin Conductance Scoring

Respiration and skin conductance were recorded using an MP150 BIOPAC system and an EDA100C amplifier. Pre‐gelled BIOPAC EL507 self‐adhesive electrodes placed on the hypothenar and thenar areas of the participants' non‐dominant hand were used to record SCR. DMDX 6.1.8.2 software (Forster and Forster 2003) was used for programming and data recording. SCRs were scored manually and only unconditioned responses (URs) are examined in this re‐analysis. URs were recorded as the highest skin conductance response beginning in the interval starting from 1 s post US onset until 4 s following US offset. URs were scored as the difference between the peak and the trough of the response. If no response began within this time interval, 0 was recorded.

#### 1.3.4 Average Film and Shock Valence Ratings

Participants rated the intensity and pleasantness of the film clips that were used as USs as well as the shock post‐extinction. Two 9‐point Likert scales for each US were used, with 1 = “very mild” to “very unpleasant” and 9 = “very intense” to “very pleasant” respectively. Ratings of pleasantness were reverse‐coded, and an average score was calculated per participant for the film clips.

#### 1.3.5 Intrusive Memory Diary

The intrusive memory diary assessed the number, distressfulness, and content of the intrusive memories experienced in the lab setting, and in the participants' external world for the follow ups. For both number and distress, participants provided a single response: a number for the number of experienced intrusive memories and a number on a 1 to 4 Likert scale indicating the distress of the intrusive memories, where 1 was “not distressing at all” and 4 was “extremely distressing”. Participants were asked to report the content of the intrusive memories in a free‐text box. Participants were asked to report enough concise detail so that the intrusive memory could be identified by research staff, and to provide brief content for each experienced intrusive memory (e.g., “fire extinguisher and face”). The intrusive memory diary was administered after reacquisition and digitally distributed to participants for the next four days. The researchers checked that participants understood how to report their intrusive memories properly during the experiment.

Two independent raters (J.A. and G.N.L) classified the intrusive memory content based on a classification system consisting of 18 subcategories. These subcategories fell under three broad categories, which are reported in Table S1 and consisted of conditioning stimuli (background colors, CSs), longer trauma film clips, and USs (aversive, neutral, and shock). Reported intrusive memories that did not match any of these categories were not included in the analyses. Note that for this article, only the intrusive memories matching the USs were used for the analyses.

### 1.4 Design and Procedure

As only acquisition data is assessed in this report, the design in this re‐analysis consisted only of two within‐subjects' factors. The 3(CS: CS_1_+, CS_2_+, and CS−) × Trial design tested the original hypotheses relating to the influence of shock on electrodermal responding. The CS_1_+ was followed by a shock paired with the film clips during the acquisition phase, whereas the CS_2_+ was paired with the film clips alone. The CS− was paired with neutral film clips. All film clips were presented for 16‐s. Laboratory sessions occurred between 11:00 am and 6:00 pm in‐line with research pertaining to salivary cortisol levels (De Lacerda et al. 1973). After informed consent was ascertained, participants watched the 10‐min trauma film. As instructions prior to trauma film exposure can influence participants' experiences and intrusive memory outcomes (Herzog et al. 2022), the experimenter took care not to prime participants, only informing them that the 10‐min film would contain violent media from films and the news and of the ability to withdraw if the film was too distressing. The possibility of intrusive memories was not disclosed to participants. A shock work‐up was then conducted, in which the intensity was increased incrementally by 0.1 mA until participants reached a threshold of “unpleasant, but not painful.”

During habituation, each CS was presented four times without the US (counterbalancing was applied). In acquisition, the CS_1_+ and CS_2_+ were both presented four times each, and the CS− (CS paired with a neutral film clip) was presented eight times, with all CSs reinforced 100% of the time. The CS duration was 6 s, with 10–14 s of intertrial intervals separating clip offset and CS onset. Next came extinction, which involved 5 presentations each of the CS_1_+, CS_2_+, and CS−, with no US reinforcement. Post‐extinction, participants were introduced to the phenomenon of intrusive memories and were informed about their spontaneous and involuntary nature. They were given the intrusive memory diary to be completed post‐reacquisition and were directed to record intrusive memories experienced during renewal and reacquisition. Following that, renewal was conducted, which included 4 CS_1_+, 4 CS_2_+, and 4 CS− with no USs. Next, reacquisition occurred, with 2 CS_1_+, 2 CS_2_+, and 2 CS− with US reinforcing 50% of the time. After reacquisition, participants were asked to complete the intrusive memory task. Finally, participants were informed about the digital follow‐ups and were asked to complete them for the next four days. Follow‐ups were sent to their email addresses at 5:00 pm each evening, and they were asked to retrospectively report how many intrusive memories of experimental stimuli they had experienced throughout the day, as well as what the content of their intrusive memories had been.

For this re‐analysis, only data from the intrusive memory diaries and the acquisition phase of fear conditioning are examined.

### 1.5 Statistical Analysis

US film and shock pleasantness as well as intensity ratings were compared using univariate ANOVAs. A 2(CS: CS_1_+, CS_2_+) × 4(Trial: 1–4) repeated measures ANOVA was performed to test the effect of unique US presentations on URs. To test the hypothesis that delayed URs might occur in response to the film clips, averaged skin conductance levels were visualized using *ggplot2* in R across the 16 s of the film clips (in addition to the CS interval) and assessed between film clip only and film clip plus electric shock USs. This was conducted separately for each of the four clips and was also repeated for unconditioned SCRs to the neutral film clips that followed the CS− (reported in the Figure S1). The averaged response during CS− URs is visualized on each panel presented in the main text. For completeness, range‐corrected data was also visualized and analyzed separately. Range correction was performed by dividing each SCR by the highest SCR in each participant's UR dataset. Differences between US conditions for each clip were also statistically assessed using polynomial contrasts, using the *lm* and *poly* functions in R. Linear, quadratic, cubic, and quartic models were compared for best fit using Akaike Information Criteria (AIC) and Bayesian Information Criteria (BIC). The best fit model was assessed and compared statistically between groups. *R* ^2^ was also computed as a measure of effect size.

Conditioned response and unconditioned responses were correlated using Pearson's correlation coefficient (*r*). The unconditioned response of each trial was correlated with the conditioned response of the preceding trial of the same conditioned stimulus type. These correlations are visualized in the results section.

Finally, zero‐inflated Poisson regression models for count data using maximum likelihood estimation were performed using the *zeroinfl* function in the *pscl* package in R. The predictor variable was the maximum SCR response beginning within the interval of 1–17 s following US onset, and the dependent variable was the intrusive memory load reported by participants for each individual film clip. Intrusive memory load is calculated as the number of reported intrusive memories multiplied by the reported distress of the intrusive memories. These analyses were repeated separately for each film clip as well as for intrusive memory reports that were recorded in the laboratory or at home. Note that the intrusive memory tests were completed with the combined Experiment 1 and Experiment 2 data. Tests were Bonferroni corrected, with a corrected significance value of *α* = 0.00625. Intrusive memory load was assessed with participant sex as a factor. These results are reported in the Supporting Information.

## 2 Experiment 2

### 2.1 Methods

While Experiment 1 used a within‐subject design to test the effect of adding a shock US on SCRs to the film clip US and intrusive memories, Experiment 2 used a between‐subjects design with only one CS+. In this experiment, the CS+ in one group (Film+Shock group) was followed by a film clip and a shock, whereas in the Film group the CS+ was only followed by a film clip.

### 2.2 Participants

Ethical approval for Experiment 2 was received from Queensland University of Technology (QUT) Human Research Ethics Committee. Participants were recruited using the same methods as in Experiment 1. Additionally, Experiment 2 shared exclusion criteria with Experiment 1, with the addition of further exclusionary measures including cannabis use within the past month, as this study aimed to assess the effect of endogenous cannabinoids measured in hair and saliva on fear conditioning. A total of 61 participants were recruited, with one withdrawing in the acquisition phase, leaving a total of 60 participants in the final sample, consisting of 15 males and 45 females (*M*_age_ = 21.24, SD = 4.66).

### 2.3 Materials and Measures

#### 2.3.1 Trauma Film

The trauma film utilized in Experiment 1 was used again in Experiment 2.

#### 2.3.2 Neutral Film Clips

The neutral film clips included in this study were the same scenes discussed in Experiment 1, which were ascertained from the “Mixed emotions film library” (Samson et al. 2016).

#### 2.3.3 Fear Conditioning

##### 2.3.3.1 Unconditioned and Conditioned Stimuli

The fear paradigm utilized in Experiment 2 included habituation, acquisition, extinction, renewal, and reacquisition. Like Experiment 1, CSs consisted of geometric shapes, namely triangles, circles, and squares, with the shapes counterbalanced between roles of CS+ and CS−. A Digitimer DS7A and a concentric shock electrode were used. The CS− was one of the shapes paired with a 16‐s neutral film clip and was used as a safety signal within the acquisition and reacquisition stages. The USs used were the same four 16‐s clips used in Experiment 1. CS+ and CS− presentation was counterbalanced, with CS+ occurring on the first trial of acquisition for 50% of participants.

##### 2.3.3.2 Contextual Renewal

As Experiment 1 found no difference between complex and simple background contexts (Lam et al. 2024), Experiment 2 only used the pink (RGB 255128192) and blue (RGB 0128255) background colors, which were counterbalanced as contexts A and B. As in Experiment 1, context A was presented during habituation, acquisition, renewal, and reacquisition, and context B was presented only during extinction.

##### 2.3.3.3 Equipment and Skin Conductance Scoring

The physiological equipment used in Experiment 2 is the same as that used in Experiment 1. SCRs were scored using a semi‐automated scoring program that has been shown to have high agreement with manual scoring. This program allows scoring of first, second, and third interval responses. In this report, we only examine the URs from this data, as for Experiment 1.

#### 2.3.4 Average Film and Shock Valence Ratings

Participants provided an overall rating of the intensity and pleasantness of the film clips that were used as USs as well as the shock post‐extinction. Two 9‐point Likert scales for each US were used, with 1 = “very mild”/“very unpleasant”, and 9 = “very intense”/“very pleasant,” respectively. Ratings of pleasantness were reverse coded, and an average score was calculated per participant for the film clips.

#### 2.3.5 Intrusive Memory Diary

The intrusive memory diary administered to participants in Experiment 2 was the same as that used in Experiment 1.

### 2.4 Design and Procedure

The experiment focused on the between‐subjects factor: 2(US: Film+Shock, Film) and included the within‐subjects' factors of CS, 2(CS: CS+, CS−) and Trial. The US differed across the two experimental conditions where group Film+Shock experienced a shock in addition to a film clip throughout acquisition, whereas group Film experienced only the film clip US. The laboratory sessions were conducted between 10:30 am and 5:00 pm. Upon participant arrival, informed consent was obtained, and all participants viewed the 10‐min trauma film. Following exposure to the longer film, physiological equipment was set up and a baseline recording was taken. Next, each participant (including those in group Film) was administered a shock workup, reflective of the workup discussed in Experiment 1. The shock level established in the workup remained constant throughout the experiment for group Film+Shock, but this was the last time that group Film experienced the shock. During habituation, the CS+ and CS− were individually presented four times for 6 s at a time. Participants moved on to acquisition, during which both the CS+ and CS− were presented eight times and immediately reinforced 100% of the time with the respective US. Extinction followed, with the CS+ and CS− being presented six times each without their corresponding US. The researcher then informed participants of the nature of intrusive memories, like in Experiment 1. Next was the renewal phase by which the unpaired CS+ and CS− were presented each individually four times, followed by the reacquisition phase, where the CS+ was presented eight times and the CS− was presented eight times, with a 50% reinforcement rate for both CSs. Once these phases ended, participants were instructed to complete the intrusive memory diary. Following completion of the form, participants were informed of the digital follow‐ups required to be filled out that night and the following three nights. Like in Experiment 1, the intrusive memory diary was sent to participants each night at 5:00 pm for them to complete retrospectively.

For this re‐analysis, only the URs from acquisition and the intrusive memories in relation to these URs were assessed.

### 2.5 Data Analysis

US film and shock pleasantness as well as intensity ratings were compared using univariate ANOVAs. An 8(Trial: 1–8) × 2(Group: Film+Shock, Film) repeated measures ANOVA was performed to test the effect of Group allocation on URs. This analysis was only conducted for the CS+. To answer the hypothesis that delayed URs might occur during film clip only USs, averaged skin conductance levels were visualized using *ggplot2* in R across the 16 s of the film clips (in addition to the CS interval) and between the Film+Shock and Film groups. This was conducted separately for each of the four clips (both clip US presentations were aggregated since each film clip was presented twice during acquisition) and was also repeated for unconditioned SCRs to the neutral film clips that followed the CS− (reported in the Figure S2). The averaged response during CS− URs is visualized on each panel presented in the main text. The difference in SCR between the first and second US presentation for each film clip is also visualized in Figure S3. For completeness, range‐corrected data was also visualized and analyzed separately. Range correction was performed by dividing each SCR by the highest SCR in each participant's UR dataset. Differences between groups for each clip were also statistically assessed using polynomial contrasts, using the *lm* and *poly* functions in R. Linear, quadratic, cubic, and quartic models were compared for best fit using AIC and BIC. The best fit model was assessed and compared statistically between groups. *R* ^2^ was also computed as a measure of effect size.

Conditioned response and unconditioned responses were correlated using Pearson's correlation coefficient (*r*). The unconditioned response of each trial was correlated with the conditioned response of the preceding trial of the same conditioned stimulus type. These correlations are visualized in the results section.

Finally, zero‐inflated Poisson regression models for count data using maximum likelihood estimation were performed using the *zeroinfl* function in the *pscl* package in R. The predictor variable was the maximum SCR response beginning within the interval of 1–17 s post US onset for both film clip US presentations, and the dependent variable was the intrusive memory load reported by participants for each individual film clip. Intrusive memory load is calculated as the number of reported intrusive memories multiplied by the reported distress of the intrusive memories. These analyses were repeated separately for each film clip as well as for intrusive memory reports that were recorded in the laboratory and at home. Note that the intrusive memory tests were completed with the combined Experiment 1 and Experiment 2 data. Tests were Bonferroni corrected, with a corrected significance value of *α* = 0.00625. Intrusive memory load was assessed with participant sex as a factor. These results are reported in the Supporting Information.

#### 2.5.1 Prototypical UR to a Shock US

We further wanted to compare the difference between a shock‐only US and the USs that involved film clips. An additional dataset of 20 healthy participants (*N* = 20) was re‐analyzed from a randomly selected subset of an existing publication (Ney, Nichols et al. 2023). Participants in this study were only ever exposed to a shock US after viewing a fish, bird, or a frog CS+ that was presented for 6 s on the same‐colored background context (i.e., pink or blue) as in the previously described two Experiments. Skin conductance levels from all eight CS+ trials during acquisition (the same number as the current experiments) from these participants were averaged and used as a prototypical UR that could be compared against skin conductance levels from film clip alone and film clip in addition to shock USs. Details on this study can be found in Ney, Nichols et al. (2023). The only notable difference in this study was that participants had not viewed the trauma film prior to beginning conditioning, never saw any trauma film clips, and did not provide saliva samples.

Averaged skin conductance level to the shock‐only US from this group of participants was visualized using *ggplot2* in R against the skin conductance levels during film clip only and film clip plus shock USs from Experiment 2. In addition to this analysis, data from the larger study (*n* = 114) reported in Lipp et al. (2024), of which Ney, Nichols et al. (2023) is just a subset, were re‐analyzed to assess the relationship between CRs and URs when the US was a shock only. The exact design specifications for this study during acquisition are identical to Ney, Nichols, and Lipp. Further details on the study can be found in Lipp et al.

## 3 Results

### 3.1 Subjective Perceptions of Shock Versus Film Intensity and Pleasantness

Participants' self‐reported ratings of both shock and film intensity and pleasantness. In Experiment 1, trauma film clips were rated as significantly less pleasant (*p* < 0.001, ηp^2^ = 0.13) and significantly more intense (*p* < 0.001, ηp^2^ = 0.14) than the shock. In Experiment 2, there was no difference reported in the perceived shock and film clip pleasantness (*p* = 1.0, ηp^2^ = 0.00) or intensity (*p* = 0.470, ηp^2^ = 0.02). The average shock intensity was higher in Experiment 2 (*M* = 85.26 mA) compared to Experiment 1 (*M* = 65.31 mA), *t*(140) = 2.66, *p* = 0.009. The subjectively rated shock intensity was higher in Experiment 2, *t*(120) = 2.03, *p* = 0.045, and rated shock pleasantness was lower in Experiment 2, *t*(114) = 2.09, *p* = 0.039.

### 3.2 Effect of Shock Presence on Unconditioned Skin Conductance Responses

#### 3.2.1 Experiment 1

**FIGURE 1:**
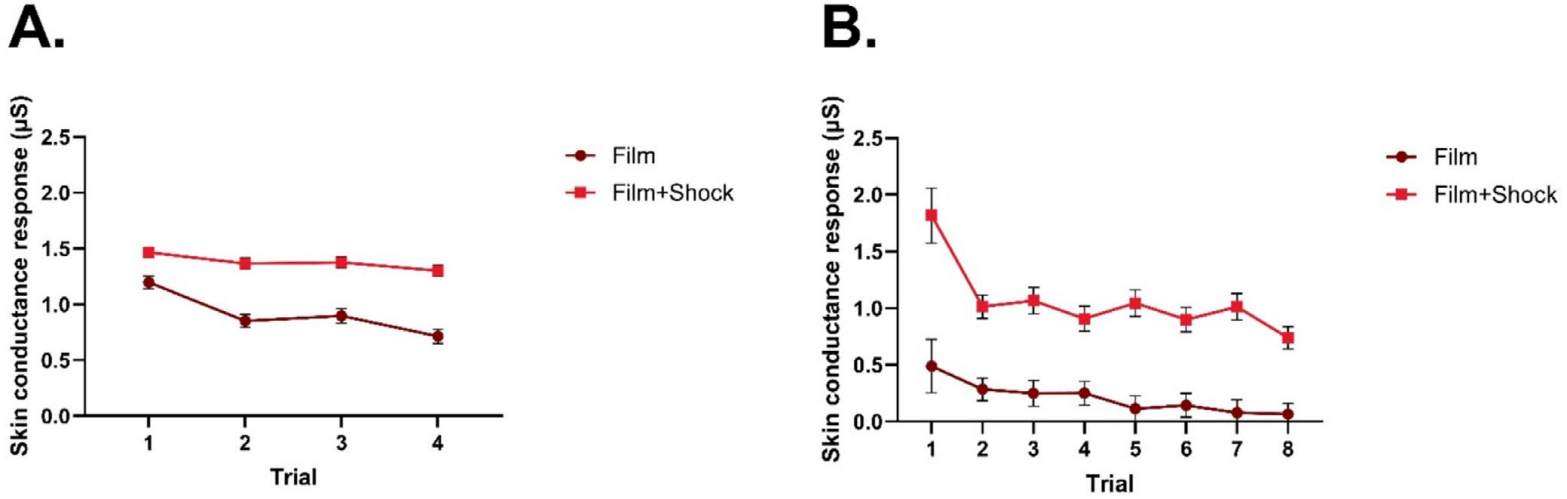
Unconditioned responses across trials of acquisition in the two Experiments. Panel A is Experiment 1, which used a within‐subjects design. Panel B is Experiment 2, which used a between‐subjects design. Error bars are standard error.

For Experiment 1, there were significant effects of CS: *F*(1, 82) = 109.62, *p* < 0.001, Trial: *F*(3, 246) = 29.58, *p* < 0.001, and CS × Trial: *F*(3, 246) = 7.89, *p* < 0.001. URs were significantly larger in response to the Film+Shock compared to the Film trials (*p* < 0.001) and the interaction effect revealed that URs reduced significantly faster across trials in Film compared to Film+Shock trials (Figure 1). When data were range‐corrected, there were significant effects of Trial, *F*(3, 246) = 41.38, *p* < 0.001, CS: *F*(1, 82) = 161.33, *p* < 0.001, and Trial × CS: *F*(3, 246) = 4.47, *p* = 0.004. The Shock CS had significantly higher URs across the phase, and this difference grew larger over time (see Figure S4A).

#### 3.2.2 Experiment 2

For Experiment 2, there were significant effects of Trial, *F*(7, 406) = 11.62, *p* < 0.001, Group: *F*(1, 58) = 36.59, *p* < 0.001, and Trial × Group: *F*(7, 406) = 2.79, *p* = 0.008. As in Experiment 1, URs were significantly larger in the Film+Shock compared to the Film group (*p* < 0.001). The Trial × Group interaction reflected a significantly higher reduction in URs after trial 1 in the Film+Shock group, whereas the Film group did not have such a large reduction in responding after the first trial (Figure 1B). When data were range‐corrected, there were significant effects of Trial, *F*(7, 399) = 16.86, *p* < 0.001, Group: *F*(1, 57) = 42.39, *p* < 0.001, but not Trial × Group: *F*(7, 399) = 1.90, *p* = 0.068. The Shock group had significantly higher URs across the phase, but this did not differ significantly between Groups over time (see Figure S4B).

### 3.3 Effect of Shock on Skin Conductance Response Timing After US Presentation

To test the hypothesis that skin conductance responses to film clips were delayed due to the prolonged 16‐s film clip duration (compared to 48‐ms shock), we visualized average skin conductance levels following CS offset separately for shock and non‐shock reinforced trials, which were further separated for each specific film clip.

**FIGURE 2:**
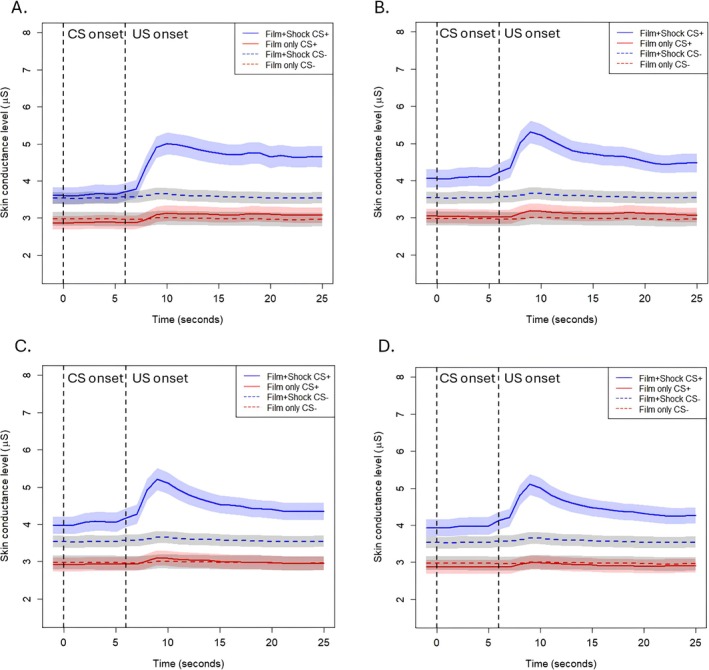
Skin conductance levels during the conditioned stimulus and immediately following unconditioned stimulus presentation during acquisition of Experiment 1. Panel A is the Irreversible clip, Panel B is the Kitchen accident clip, Panel C is the Cow clip, and Panel D is Hostel. This experiment used a within‐subjects design. CS onset = conditioned stimulus onset. US onset = unconditioned stimulus onset (coincides with CS offset). The Film only CS− is the neutral conditioned stimulus and neutral unconditioned stimulus response averaged across all neutral stimuli for the Film only group. The Film+Shock only CS− is the neutral conditioned stimulus and neutral unconditioned stimulus response averaged across all neutral stimuli for the Film+Shock group. The film clip lasted for the 16 s following US onset. Error bands are standard error of the mean.

**FIGURE 3:**
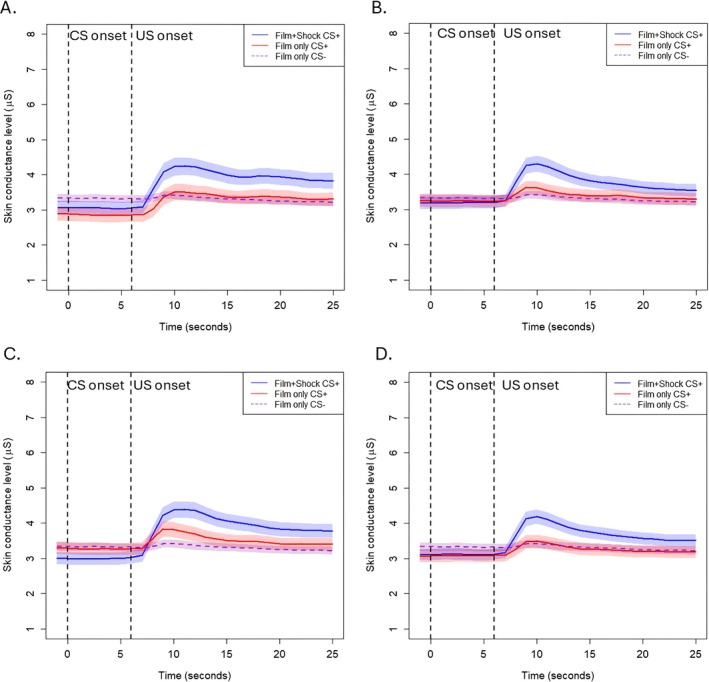
Skin conductance levels during the conditioned stimulus and immediately following unconditioned stimulus presentation during acquisition of Experiment 2. Panel A is the Irreversible clip, Panel B is the Kitchen accident clip, Panel C is the Cow clip, and Panel D is Hostel. This Experiment used a between‐subjects design. CS onset = conditioned stimulus onset. US onset = unconditioned stimulus onset (coincides with CS offset). The Film only CS− is the neutral conditioned stimulus and neutral unconditioned stimulus response averaged across all neutral stimuli. The film clip lasted for the 16 s following US onset. Error bands are standard error of the mean.

**FIGURE 4:**
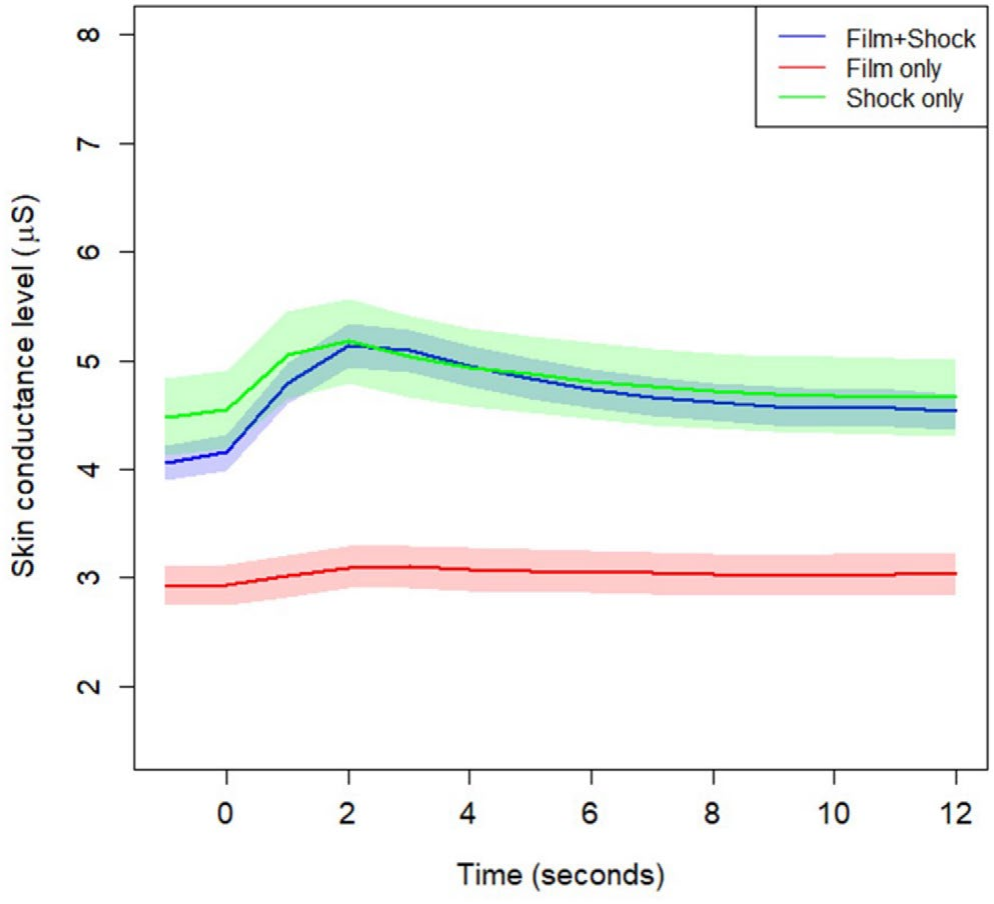
Skin conductance levels during and immediately following unconditioned stimulus presentation to a shock alone when compared to a clip plus shock and clip alone. Film+Shock and Film only responses are averaged responses to aversive unconditioned stimuli from Experiment 2, which used a between‐subjects design. Error bands are standard error of the mean.

As is visualized in Figures 2 and 3, there were no differences in the timing of SCRs between shock and non‐shock reinforced trials, either in the within‐subjects design of Experiment 1 (Figure 2) or the between‐subjects design of Experiment 2 (Figure 3). Rather, these figures show that participants simply have much larger URs when the CS is followed by a shock. The responses to the shock and film clip US were similar to those to a shock alone, which was obtained from a different sample (Figure 4), suggesting that the large SCRs following shock plus film clip USs were driven largely by the shock. For both experiments, there was slower recovery of skin conductance level following the Hostel clip, when compared to the other clips. Figures S5–S8 illustrate the individual responses to each aversive US for Experiments 1 and 2.

Differences in SCRs between the US types were also assessed using polynomial contrasts. For Experiment 1, the best fit polynomial models—assessed by AIC and BIC—were the cubic models. There was no difference in the cubic model fit between CS1+ and CS2+ in response to the Hostel clip (*p* = 0.203), though there were differences in the shape of the responses to Cow (*p* = 0.022), the Kitchen Accident (*p* = 0.050), and Irreversible (*p* = 0.048). However, these models accounted for very minimal amounts of variance (adjusted *R* ^2^ = 0.03 for all significant effects), and visually it is evident that these effects were driven by the higher response to shock rather than higher responses in the no shock CS+ at a later point in the US presentation. For Experiment 2, AIC and BIC revealed that responses to USs were best fit to linear models, with the exception of responses to the Hostel clip, which was best fit to a cubic model. In all cases, there was no difference in the shape of the response between Groups (all *p*s > 0.11).

Visualization of the responses to the neutral film clips that reinforced the CS− during acquisition similarly suggested no differences between the groups or film clips (see Figures S1 and S2), except that in Experiment 2 (between group design, Figure S2) there was a higher overall skin conductance level (but not skin conductance response) in the group that was receiving the shock.

### 3.4 Relationship Between Conditioned and Unconditioned Responses

**FIGURE 5:**
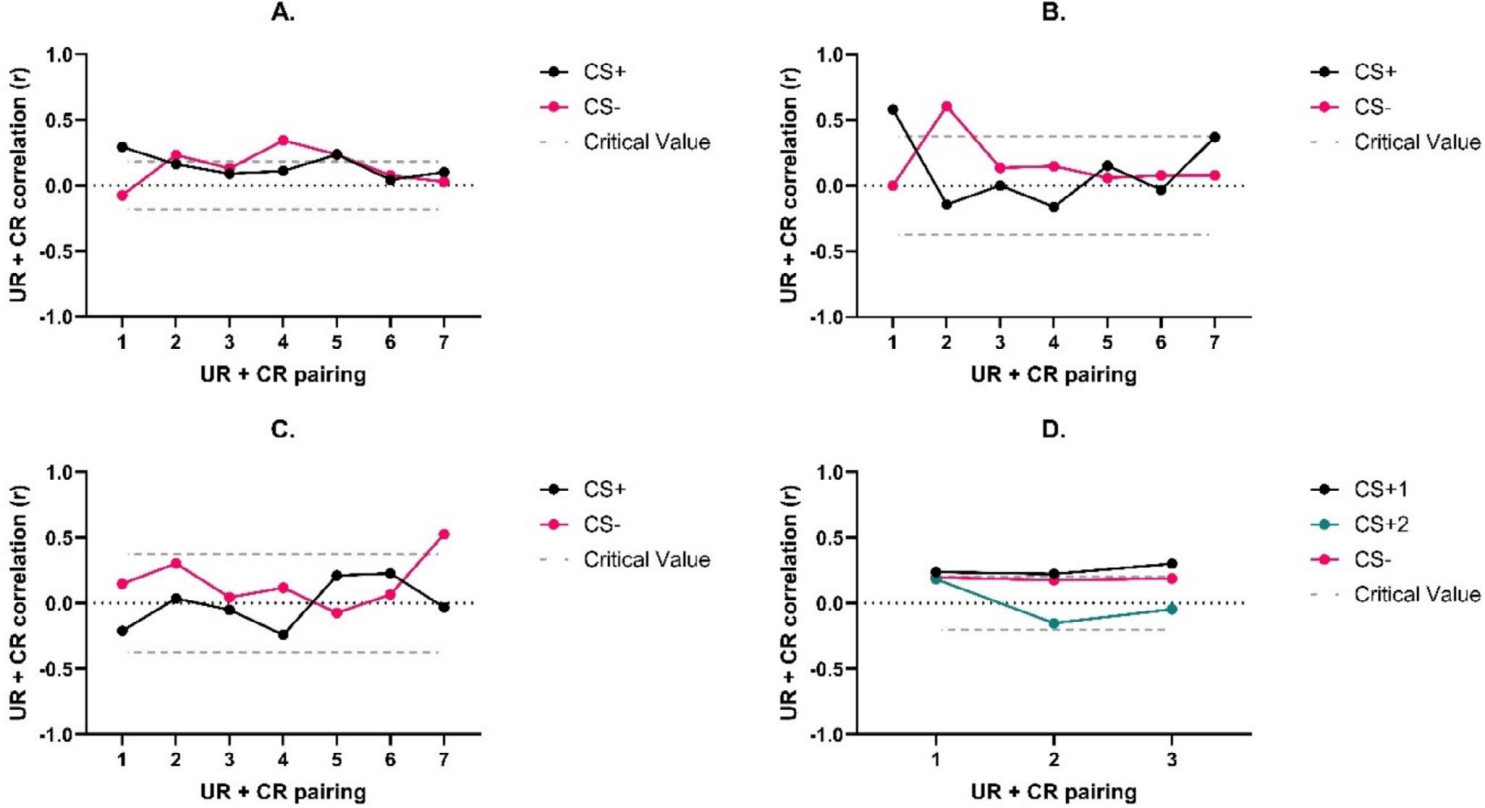
Pearsons correlations (r) between corresponding unconditioned responses and conditioned responses in each condition of the experiments. Panel A is the Shock only group. Panel B is the Film+Shock group from Experiment 2. Panel C is the Film group from Experiment 2. Panel D is from Experiment 1, where CS + 1 is Film+Shock and CS + 2 is Film only. The *y* axis is the correlation between corresponding unconditioned response (UR) and conditioned response (CR) pairs. The *x* axis is the UR trial number. UR‐CR pairs include the UR from the trial preceding the paired CR. The Critical Value lines represent the upper and lower significance levels for each analysis at *p* < 0.05.

Pearson's correlations were conducted to test the correlation between conditioned and unconditioned responses in each condition of the experiments. These correlations are reported in Figure 5.

### 3.5 Association Between Responses to Individual Clips and Content of Reported Intrusive Memories

Finally, we conducted an analysis measuring the association between the maximum SCR during each individual film clip presentation (as a US) and the intrusive memory load (number of intrusive memories multiplied by intrusive memory distress) reported by participants for each specific film clip. These analyses were conducted separately for intrusive memories reported in the lab and during the follow‐up sessions. Due to the high number of intrusive memory contents reported as zero for some of the film clips, zero‐inflation models were fitted to comparisons where zero‐inflation was a better model fit compared to the count model.

**FIGURE 6:**
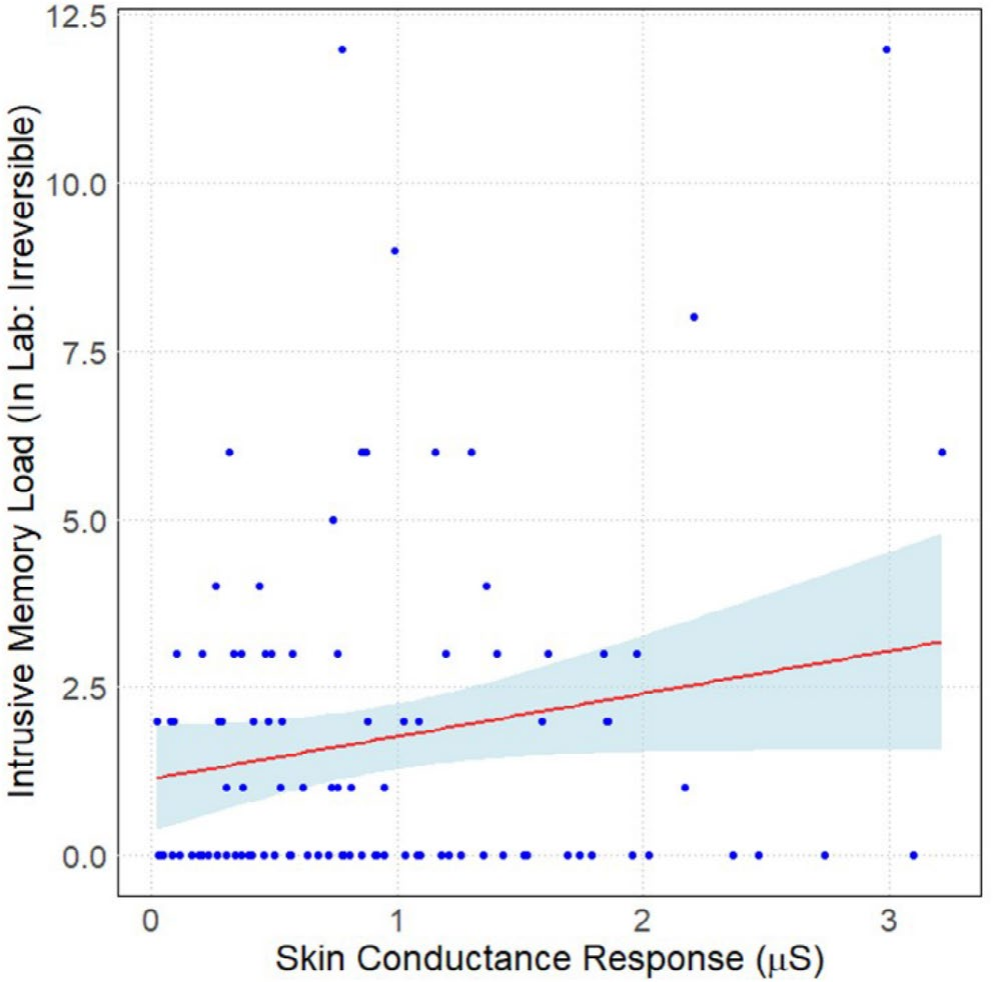
Higher unconditioned responses during the irreversible clips were associated with higher intrusive memory load during Experiments 1 and 2 laboratory session. Error band is 95% confidence interval.

There was no association between SCRs to the Hostel clip and reported intrusive memories of the Hostel clip, either reported in the laboratory (*β* = −0.01, SE = 0.17, *p* = 0.949) or in the follow‐up reports (*β* = −1.11, SE = 0.27, *p* = 0.678). No association existed between SCRs to the Kitchen Accident clip and reported intrusive memories of the Kitchen Accident clip, either in the laboratory (*β* = 0.15, SE = 0.28, *p* = 0.584) or in the follow‐up reports (*β* = −0.41, SE = 0.37, *p* = 0.273). There was no significant association between SCRs to the Cow clip and intrusive memories of the Cow clip in the laboratory (*β* = 0.13, SE = 0.25, *p* = 0.356) or in the follow‐up reports (*β* = −0.26, SE = 0.29, *p* = 0.368). Finally, there was a positive and significant association between SCRs to the Irreversible clip and intrusive memories of the Irreversible clip in the laboratory (*β* = 0.36, SE = 0.10, *p* < 0.001, Figure 6) but no significant effect in the follow‐up reports (*β* = −0.54, SE = 0.27, *p* = 0.050).

## 4 Discussion

Previous studies have reported that physiological fear conditioning in response to aversive film clips and sounds is inferior compared to fear conditioning in response to mild electric shocks. The current article explored some of the potential factors that contribute to this phenomenon. To do this, we assessed the relationship between skin conductance responses (SCRs), intrusive memories, and different types of unconditioned stimuli (USs) in two fear conditioning experiments. Our results revealed that unconditioned SCRs (URs) were smaller to aversive film clip USs compared to a US consisting of a shock in addition to an aversive clip. Despite this, participants rated the shock as equally or less aversive and intense than the aversive film clips. Visualization of the timing of URs suggested that there was no delayed response to the film only USs; however, there was some evidence of differences in responding across individual aversive film clips. Statistically, this was broadly supported, with some small differences in response shape between USs in Experiment 1. However, there was only a weak and inconsistent relationship between CRs and URs, with no relationship detectable between CRs and URs when the USs consisted only of film clips. Finally, intrusive memory frequency was associated with the size of the URs but only in one specific case. These findings contribute significantly to our knowledge of how skin conductance responds to different types of stimuli and provide new insights into experimental intrusive memory research.

There is compelling evidence in the trauma film literature that SCRs are higher when an electro‐tactile shock is added to the aversive film clips in comparison to a film‐alone US (Franke et al. 2022; Ney, Schenker et al. 2022). Our present analyses of URs further support this finding by showing that SCRs were larger not only in response to the CS associated with a shock US as previously demonstrated (Ney, Schenker et al. 2022), but also directly in response to a shock US when compared to a US that is a film clip alone. One possible account for weaker SCRs to CSs that are reinforced by film clips is trace conditioning, where longer time between CS offset and US onset causes weaker conditioning (Rescorla 1988). However, our results show that there is no delayed UR to the film clips, perhaps in response to the most traumatic aspects of the clip—and therefore unlikely trace conditioning—in two datasets that show weak electrodermal conditioning in response to film clip USs. There were no delayed responses during URs that occurred in the film only US conditions that did not occur in the shock and film US conditions, and overall, Hostel was the only clip that showed some evidence of a delayed US response. If the lack of electrodermal conditioning in these datasets was due to trace conditioning, then we would have expected an equally large, delayed UR across all clips. This was not the case. Similarly, it is possible that videos could elicit steady increases in skin conductance levels without producing typical transient SCRs. The data presented in the current article also suggested that this was not the case—the majority of participants had prominent URs beginning in the interval immediately post US onset, though there were overall smaller UR amplitudes, lower skin conductance level, and more non‐responses in the film only conditions. This could be indicative of generally lower arousal in the absence of a shock, which could interfere with subsequent reactivity during conditioning.

Despite this finding, it is critical to note that—and consistent with the findings of Ney, Nichols et al. (2023)—shocks were rated by participants as subjectively either less or equally aversive and intense compared to the film clips. This implies that the subjective experience of US aversiveness cannot account for the differences in conditioned electrodermal responding across USs, or the differences in participant arousal. Unfortunately, we did not collect online US expectancy data, so we cannot confirm whether differences in SCRs were due to differences in contingency knowledge. Future studies should repeat this experiment while collecting US expectancy. If differences in conditioned electrodermal responding remain despite no difference in US expectancy or US valence between groups receiving a shock US compared to a film clip US, we could conclude that not only does trace conditioning not account for this interesting phenomenon, but also that contingency learning and perceived US aversiveness have no influence on conditioned electrodermal responding. At this stage, our hypothesis is that conditioning when exposed to an electric shock is highly driven by a physical—rather than a psychological—reaction. The implications of this conclusion for the wider fear conditioning literature may be substantial as it may suggest that conditioning processes may be separable depending on whether the US involves physical stimulation. However, it will be important to test in the future whether the mere absence of a shock, which results in lower overall arousal and potentially lower electrodermal responsivity, is the cause of lower film clip conditioning rather than a US‐specific effect. Future studies could present unpaired shocks (like in Lipp et al. (2025)) during film‐only acquisition to determine whether higher arousal that is non‐US‐specific leads to stronger conditioning. It is also important to consider that previous trauma film conditioning experiments have not controlled for the frequency of participants' exposure to violent content, though it has been found that frequent observers of violent content may be desensitized to aversive film content (Carnagey et al. 2007; Fanti et al. 2009; O'Donohue et al. 2024) and thus potentially display less of a physiological response to films compared to a novel electric shock during conditioning, even though the film clips may be perceived as subjectively more aversive. Future studies will need to carefully control for violent content exposure and its effect on subsequent conditioning.

Although the present data allow us to rule out trace conditioning as an explanation for the lack of conditioned responding with film clip USs, we also tested whether the amplitude of CRs were associated with the amplitude of corresponding URs. This analysis was conducted because, despite there being no difference in UR timing, there was lower UR amplitude to film clip only USs. The correlations between URs and CRs were weak in conditions with shock USs and were not present in conditions with film clip only USs. These data suggest that there may be a relationship between the overall UR and CR. The design of this re‐analysis did not allow us to answer whether this possible effect was due to the type of US or just due to the amount of physiological arousal under each condition. A future study could test whether a non‐shock US that produces equivalent unconditioned responding to a shock US results in equivalent conditioned responding. However, it should be noted that the UR‐CR relationship in the shock conditions was not reliable, indicating that this may not be a stable measure to evaluate our hypotheses.

Visual analysis of skin conductance level changes during US presentations revealed some potential differences in UR timing between the individual film clips. This finding is consistent with the fact that the different clips had different events occurring at different times within the 16 s of their presentation. The most prominent difference in URs was seen for the Hostel film clip, for which a large SCR can be seen approximately 10 s into the 16 s runtime, which was an effect that was not evident for the other film clips. At approximately 9 s into the Hostel clip occurs a vivid depiction of a man having his body pierced by a power drill occurs—this timing corresponds to the large SCR observed in this clip.

We also explored the relationship between intrusive memory frequency and URs for each clip. Content analysis of the intrusive memories was performed, and we were able to categorize intrusive memories into frequencies for each clip. These results revealed an association between the intrusive memory frequency and URs for in‐laboratory reports of Irreversible, but not the other film clips. Specifically, the reported in‐laboratory intrusive memories for the Irreversible clip were more frequent for participants who displayed a larger UR to the Irreversible clip. However, there were no associations between intrusive memory frequency and UR magnitude for the other film clip USs. These findings therefore only partially support our hypothesis regarding higher SCRs being associated with greater intrusive memory frequency. Our results are somewhat in contrast to the existing literature, which has clearly shown that activity in brain areas such as the anterior insula and dorsal anterior cingulate cortex is associated with intrusive memory frequency (Miedl et al. 2024, 2020). When subjected to machine learning, brain activity can be used to predict intrusive memory frequency with moderate (68%) accuracy between participants (Clark et al. 2014). One explanation for the weakness of the association found in our study is that the relationship between skin conductance responding and brain activity is not well understood (Boucsein 2012).

A limitation of this study is the lack of record of the participants' personal trauma exposure. As per the original studies, data surrounding each person's trauma history was not collected; hence, we cannot determine if self‐report or physiological responses to the accidental or interpersonal traumatic events were influenced by previous exposure to trauma. Additionally, the combination of between‐subjects and within‐subjects designs of the experiments may have contributed to the conflicting data, particularly in regard to the subjective interpretations of the shock versus film‐alone pleasantness and intensity, as seen by the lack of a difference in Experiment 2 compared to the difference between USs in Experiment 1. Future studies should also measure US expectancy to confirm whether there is a difference in expectancy learning between film clip and electric shock USs during conditioning. Additionally, further research should dissect US content, namely whether the presence of the stimuli in everyday settings influences the relationship between SCRs and intrusive memory frequency. Our study was also limited by the use of retrospective intrusive memory assessment (i.e., a daily memory diary) rather than ecological momentary assessment, which may be a more valid measurement technique (Iyadurai et al. 2019; James et al. 2016). Moreover, the current study was a re‐analysis of data from studies not explicitly designed to test the research hypotheses. There was no experimental group that had experienced a Shock‐only US; this data was drawn from another study. Finally, the seminal trauma film conditioning studies did not present a longer trauma film prior to conditioning (Wegerer et al. 2013, 2014). While we acknowledge that our inclusion of the longer film prior to conditioning may have affected conditioning, Kunze et al. (2015) as well as unpublished data from our group (under review at *Biological Psychology*) found that conditioning was enhanced in participants who viewed a trauma film prior to conditioning, compared to controls that either viewed no film or a neutral film.

In summary, previous studies have shown that electric shocks produce stronger electrodermal fear conditioning compared to audio or video stimuli, even if the audio or video stimuli are perceived as more intense and aversive by participants. The data presented in this study rule out the possibility that in the case of video USs this is due to trace conditioning and further suggest that electric shocks might produce stronger conditioning for reasons that do not relate to feelings of psychological fear or threat. Instead, our data in this study lead us to hypothesize that an electric shock creates an autonomic reflex response associated with anticipation of tactile reinforcement, which may be an evolved preparedness for potential tissue damage. It is important to consider that the presumed learning processes occurring during fear conditioning may need to be reconceptualized if it is possible to discriminate between anticipatory responses to physical versus psychological stimuli. This is especially important to consider that most animal research uses painful electric shocks, which would therefore not be congruent with the presumed psychological processes underlying many types of human fear conditioning. Since this study presents an opportunistic reanalysis of experiments not designed to explicitly test this hypothesis, carefully planned future studies that are designed to test the impact of physical versus psychological USs on fear conditioning are needed for conclusive research on this topic. Our data also show that different video clips in some cases produce unique URs, which may depend on the specific timing of the aversive events within each film clip. Finally, more work is needed to understand the specific relationship between SCRs during film clip USs and the resulting intrusive memories reported by participants—we only find partial evidence for such a relationship in the current data.

## Author Contributions

**Lilyan Tyson:** conceptualization, data curation, formal analysis, methodology, visualization, writing – original draft, writing – review and editing. **Ruqayya Dawoodjee:** data curation, formal analysis, writing – original draft. **Joe Anderson:** data curation, formal analysis, project administration, writing – review and editing. **Ottmar V. Lipp:** conceptualization, supervision, writing – review and editing. **Gia Nhi Lam:** data curation, formal analysis, investigation, project administration, writing – review and editing. **Kalia White:** data curation, formal analysis, investigation, project administration, writing – review and editing. **Jack Cooper:** data curation, formal analysis, investigation, project administration. **Luke J. Ney:** conceptualization, formal analysis, funding acquisition, methodology, supervision, visualization, writing – review and editing.

## Conflicts of Interest

The authors declare no conflicts of interest.

## Supporting information


Data S1.

## Data Availability

Data is available upon request from the authors.
