# Supplementary material for: Fear Conditioning With Film Clip and Electric Shock Unconditioned Stimuli: What Drives Conditioned Electrodermal Responses?
Source: Psychophysiology. 2025 Jun 10;62(6):e70089. doi: 10.1111/psyp.70089 (PMC12152407; doi:10.1111/psyp.70089)
Supplement: Supplementary file 1 — Data S1. [file PSYP-62-e70089-s001.docx]

**Supplementary Material**

**Supplementary Figure 1**

*Skin conductance levels during the neutral conditioned stimulus and immediately following neutral unconditioned stimulus presentation during acquisition of Experiment 1*


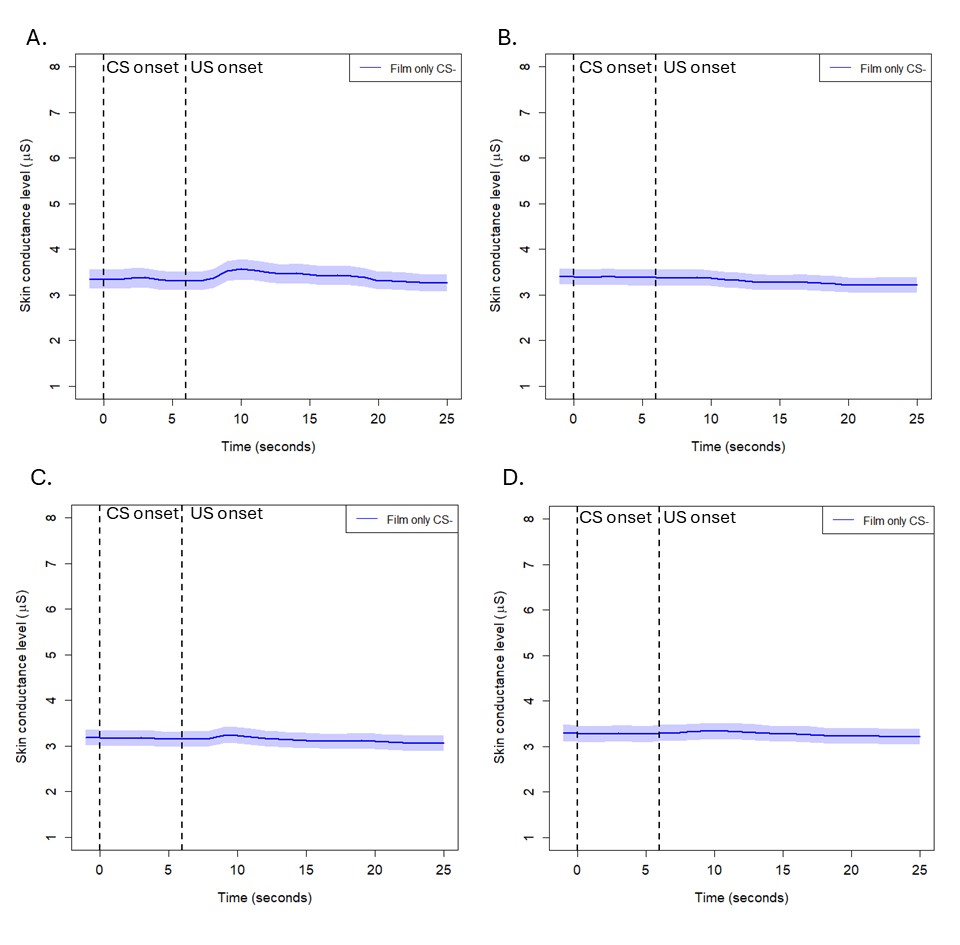


*Note:* Panel A is the Shovelling Snow clip, Panel B is the Subway Train clip, Panel C is the Riding Horse clip, and Panel D is the Café Clip. This Experiment used a within-subjects design. CS onset = conditioned stimulus onset. US onset = unconditioned stimulus onset (coincides with CS offset). The film clip lasted for the 16 seconds following US onset. Error bands are standard error of the mean.

**Supplementary Figure 2**

*Skin conductance levels during the neutral conditioned stimulus and immediately following neutral unconditioned stimulus presentation during acquisition of Experiment 2*


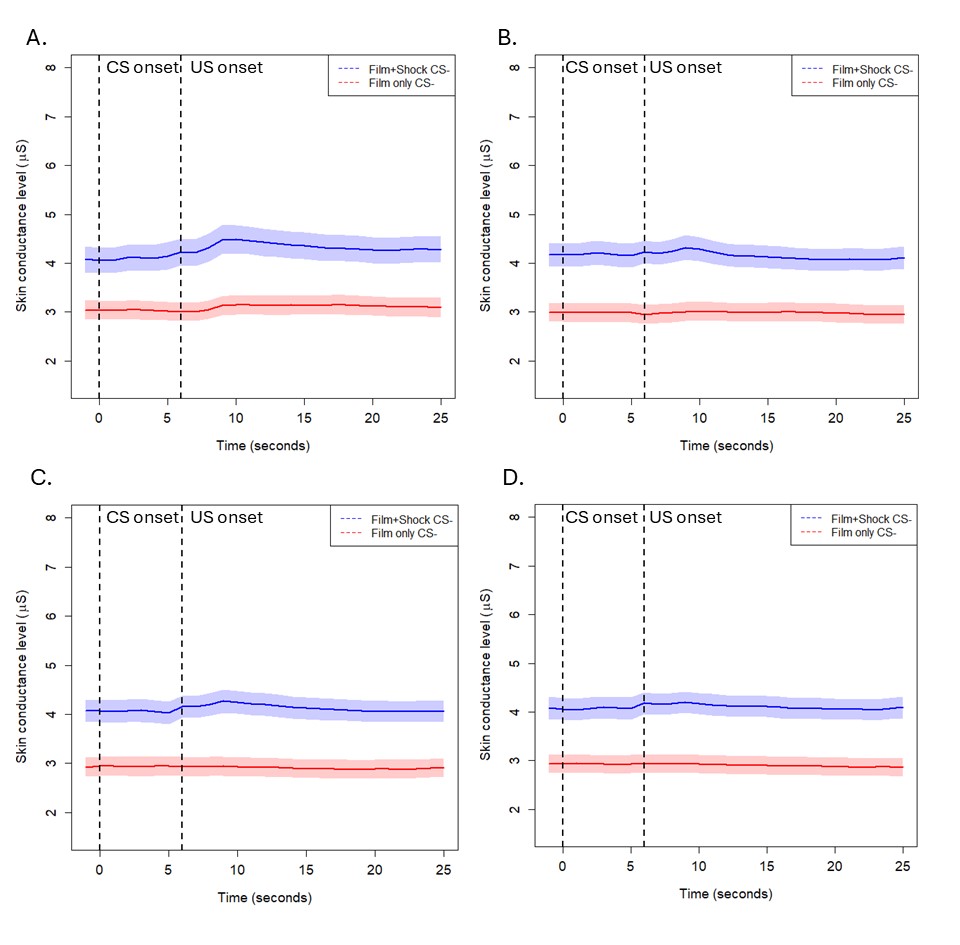


*Note:* Panel A is the Shovelling Snow clip, Panel B is the Subway Train clip, Panel C is the Riding Horse clip, and Panel D is the Café Clip. This Experiment used a between-subjects design. CS onset = conditioned stimulus onset. US onset = unconditioned stimulus onset (coincides with CS offset). The film clip lasted for the 16 seconds following US onset. Error bands are standard error of the mean.

**Supplementary Figure 3**

*
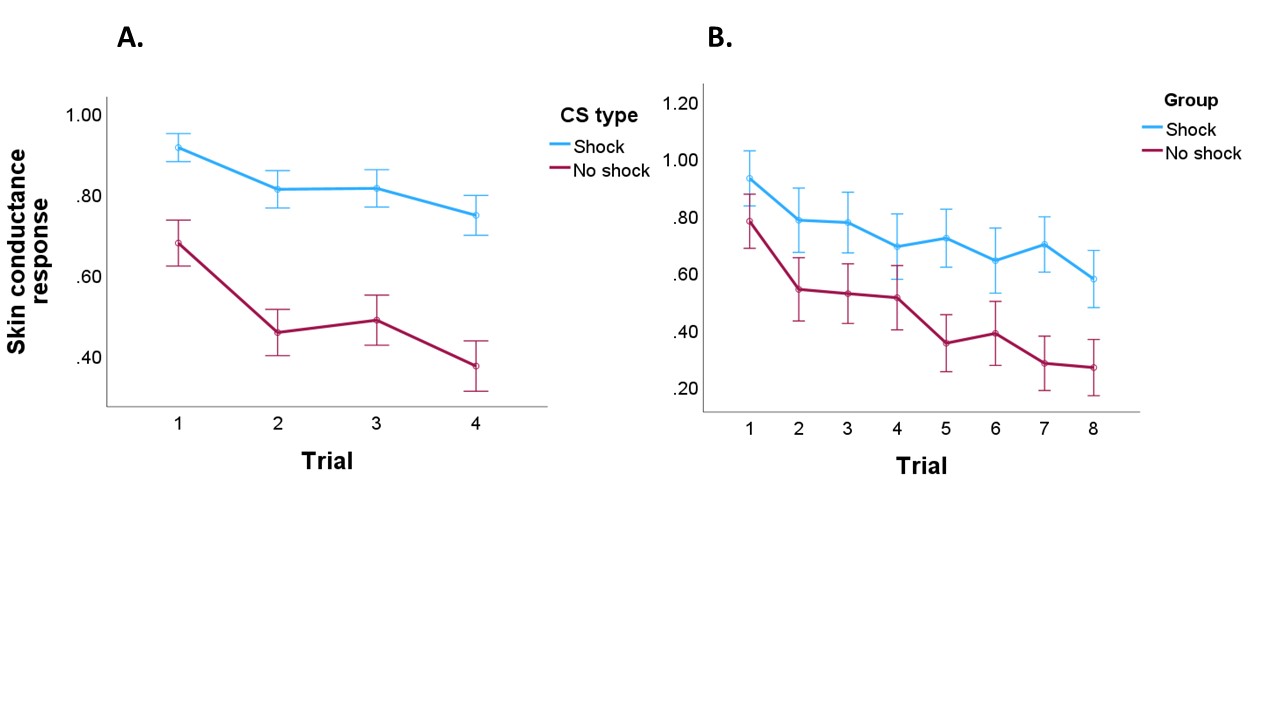
Range-corrected unconditioned responses across trials of acquisition in the two Experiments*

*Note:* Panel A is Experiment 1, which used a within-subjects design. Panel B is Experiment 2, which used a between-subjects design. Error bars are 95% confidence intervals.

**Supplementary Figure 4**

*Difference between first and second presentation of each aversive unconditioned stimulus film clip in Experiment 2*


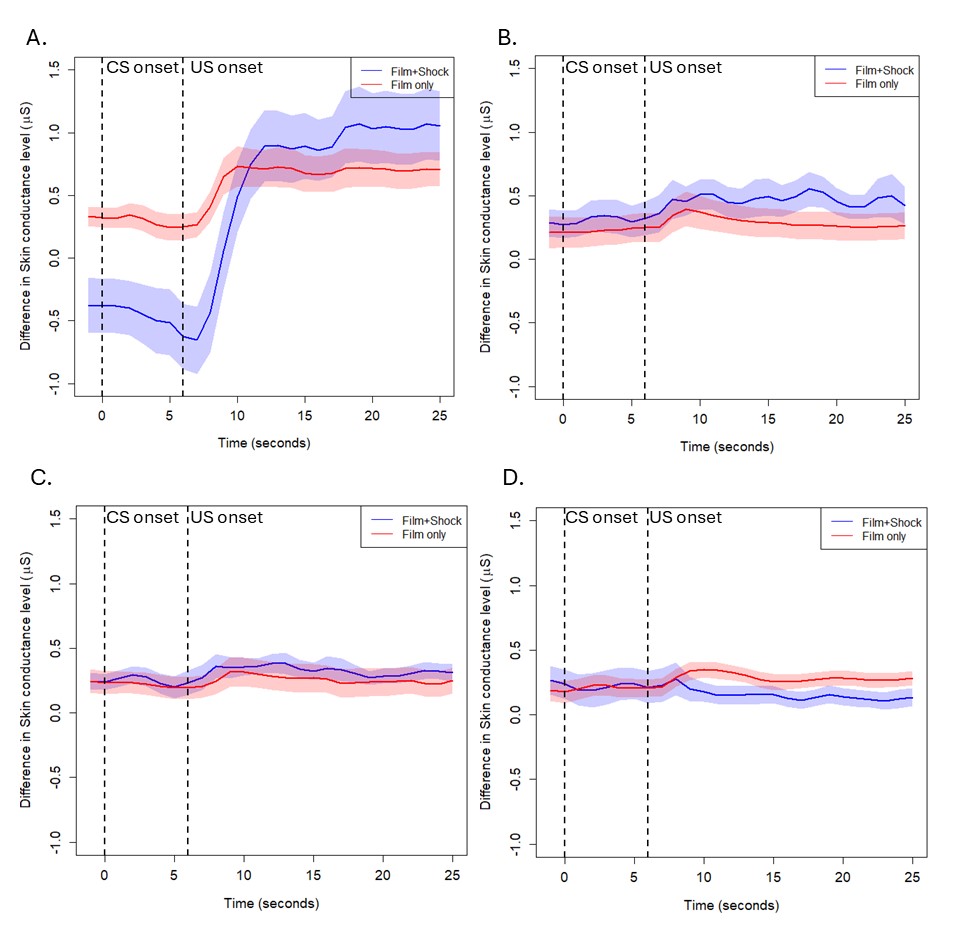


*Note:* Panel A is the Hostel clip, Panel B is the Kitchen accident clip, Panel C is the Cow clip, and Panel D is Irreversible. CS onset = conditioned stimulus onset. US onset = unconditioned stimulus onset (coincides with CS offset). The film clip lasted for the 16 seconds following US onset. Error bands are standard error of the mean.

**Supplementary Figure 5**

*Individual* s*kin conductance levels during the conditioned stimulus and immediately following Film only aversive unconditioned stimulus presentation during acquisition of Experiment 1*


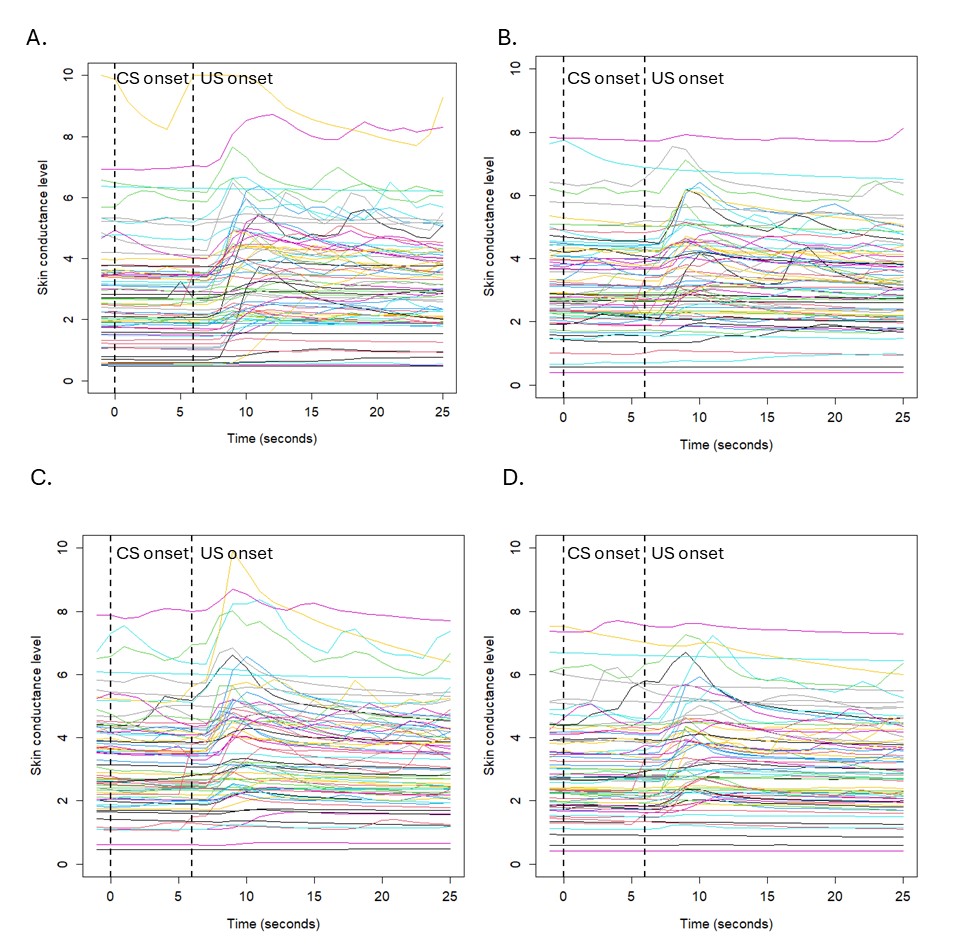


*Note:* Panel A is the Hostel clip, Panel B is the Kitchen accident clip, Panel C is the Cow clip, and Panel D is Irreversible. CS onset = conditioned stimulus onset. US onset = unconditioned stimulus onset (coincides with CS offset). The film clip lasted for the 16 seconds following US onset.

**Supplementary Figure 6**

*Individual* s*kin conductance levels during the conditioned stimulus and immediately following Shock+Film aversive unconditioned stimulus presentation during acquisition of Experiment 1*


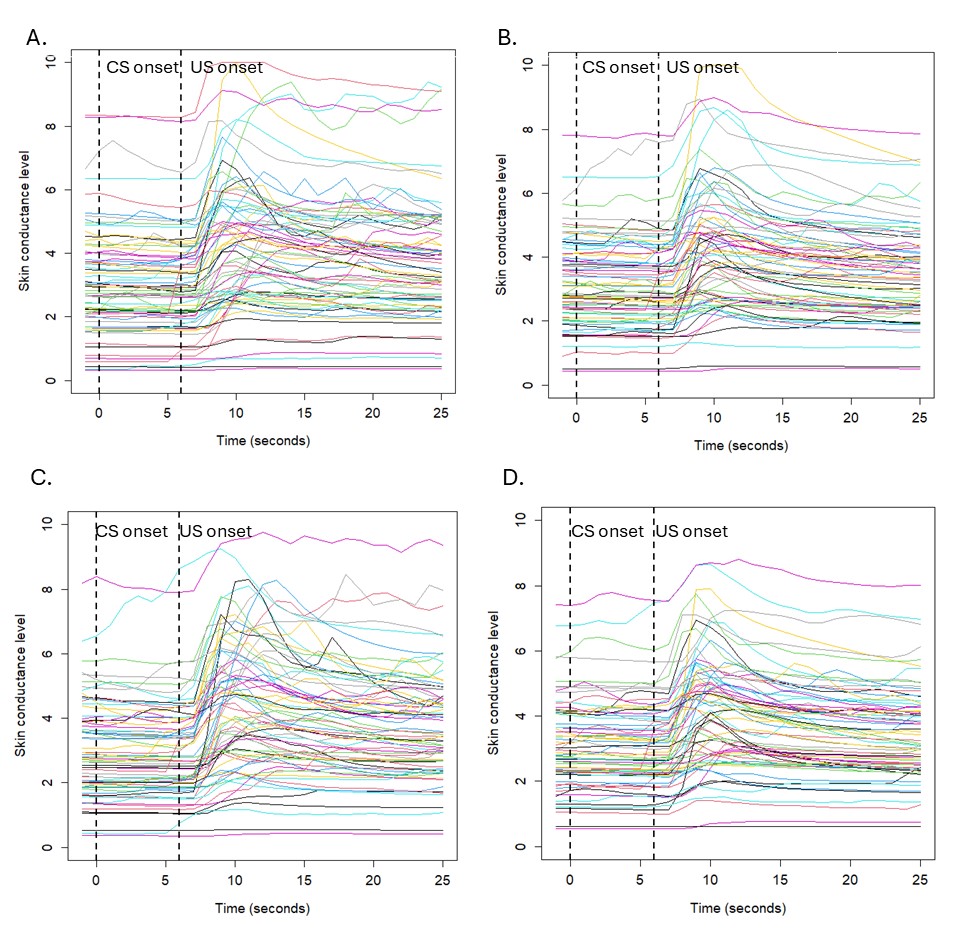


*Note:* Panel A is the Hostel clip, Panel B is the Kitchen accident clip, Panel C is the Cow clip, and Panel D is Irreversible. CS onset = conditioned stimulus onset. US onset = unconditioned stimulus onset (coincides with CS offset). The film clip lasted for the 16 seconds following US onset.

**Supplementary Figure 7**

*Individual* s*kin conductance levels during the conditioned stimulus and immediately following aversive unconditioned stimulus presentation during acquisition of Experiment 2 in the Film only group*


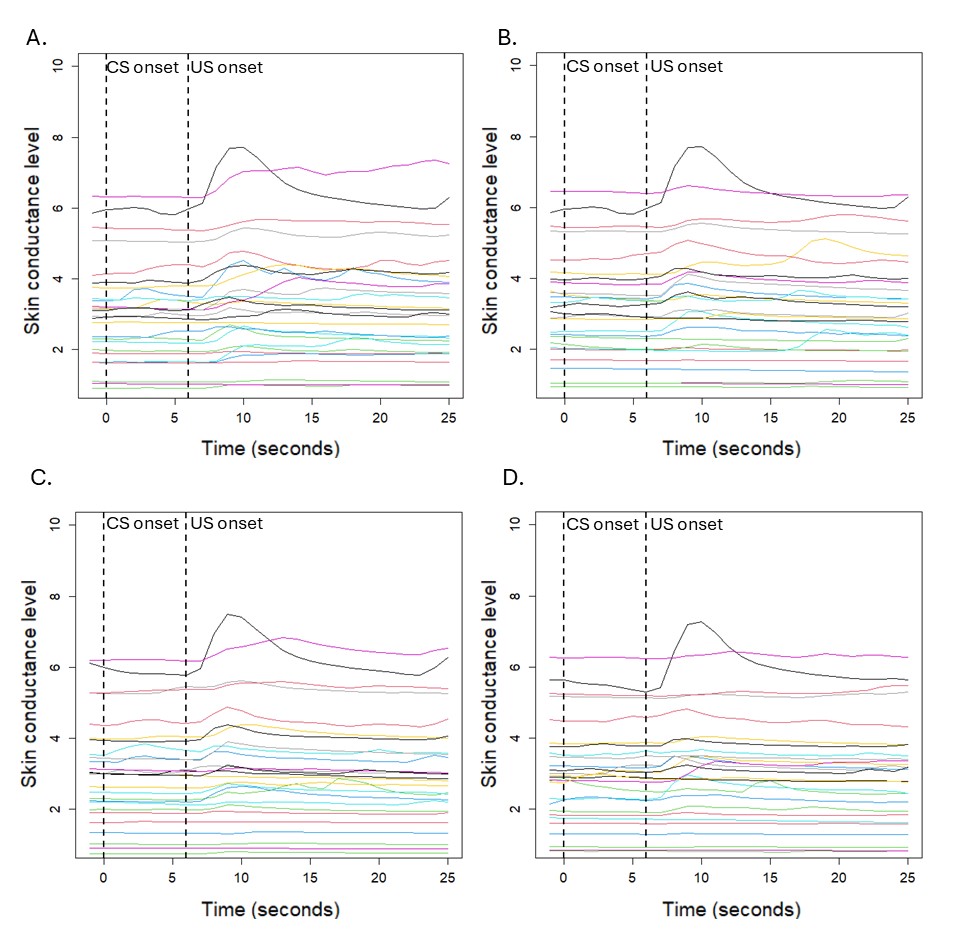


*Note:* Panel A is the Hostel clip, Panel B is the Kitchen accident clip, Panel C is the Cow clip, and Panel D is Irreversible. CS onset = conditioned stimulus onset. US onset = unconditioned stimulus onset (coincides with CS offset). The film clip lasted for the 16 seconds following US onset.

**Supplementary Figure 8**

*Individual* s*kin conductance levels during the conditioned stimulus and immediately following aversive unconditioned stimulus presentation during acquisition of Experiment 2 in the Shock+Film group*


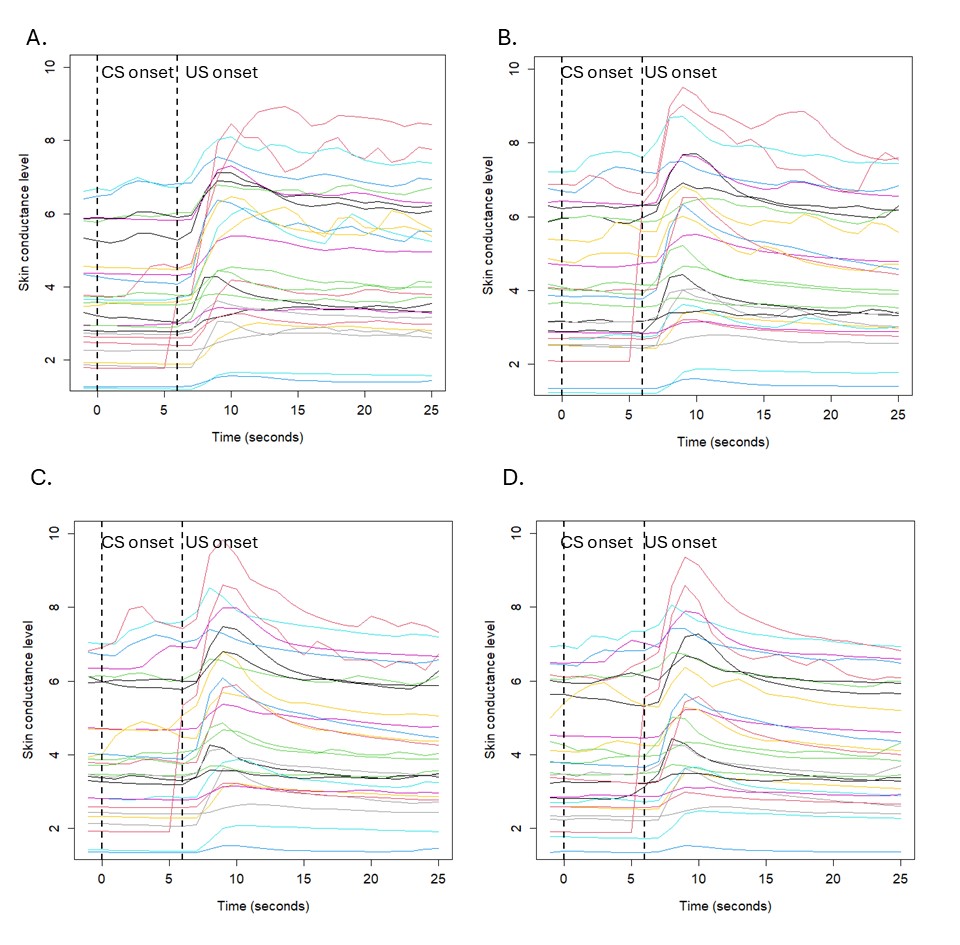


*Note:* Panel A is the Hostel clip, Panel B is the Kitchen accident clip, Panel C is the Cow clip, and Panel D is Irreversible. CS onset = conditioned stimulus onset. US onset = unconditioned stimulus onset (coincides with CS offset). The film clip lasted for the 16 seconds following US onset.

**Supplementary Table 1**

*Categories and subcategories of intrusive memory responses*

| **Category** | **Subcategory** | **Example of reported intrusive memory** |
| --- | --- | --- |
| Unconditioned Stimulus | “Hostel” | “man being drilled” |
|  | “Irreversible” | “fire extinguisher and face” |
|  | Kitchen Accident | “chef slipping in oil” |
|  | “Cow” | “texting while driving leading to accident” |
|  | Snowplow | “man plowing snow” |
|  | Café | “man sitting at café watching people” |
|  | Train | “subway train full of people” |
|  | Horse | “person riding galloping horse” |
|  | Electric shock | “feeling like I remembered the shock feeling” |
| Longer trauma film clips | “Drive” | “woman’s head being shot” |
|  | Leopard attack | “leopard biting man” |
|  | TV news mugging | “strangled by jewellery” |
| Conditioning Stimuli | Triangle | “triangle shape” |
|  | Circle | “circle” |
|  | Square | “square” |
|  | Pink colour (background) | “the colour pink” |
|  | Blue colour (background) | “blue colour” |
|  | Blank screen | “just a blank white screen” |

**Effect of participant sex on unconditioned responses**

The effect of participant sex on unconditioned responses (URs) was assessed. There was no effect of sex on URs in Experiment 1, F(1,75) = 0.11, p = .739. This lack of effect persisted in the interaction between CS type and sex, F(2,150) = 0.64, p = .531. There was no effect of sex on URs in Experiment 2, F(1,52) = 0.23, p = .633. This lack of effect persisted regardless of the interaction of sex with group, F(1,52) = 0.64, p = .429, or with CS type, F(1,52) = 1.68, p = .201.

**Effect of participant sex on intrusive memory load**

The effect of participant sex on intrusive memory load was assessed using Wilcoxon rank sum tests. This test was conducted for in laboratory intrusive memories and at home intrusive memories separately. Data from Experiments 1 and 2 were combined for these analyses. There was no effect of participant sex on intrusive memory load, W = 1717, p = .180, though females had a qualitatively higher intrusive memory load (M = 3.54, SD = 6.97 for females compared to M = 1.09, SD = 2.29 for males). Similarly, there was no significant difference between males and females on intrusive memory load, W = 1790.5, p = .099, though females had a qualitatively higher intrusive memory load (M = 5.13, SD = 6.64 for females compared to M = 3.09, SD = 4.55 for males).
